# Virtual Surface Characteristics of a Tactile Display Using Magneto-Rheological Fluids

**DOI:** 10.3390/s110302845

**Published:** 2011-03-02

**Authors:** Chul-Hee Lee, Min-Gyu Jang

**Affiliations:** Department of Mechanical Engineering, Inha University, Yonghun-dong 253, Nam-gu, Incheon 402-751, Korea; E-Mail: bomgal28@hanmail.net

**Keywords:** tactile, magnetorheological fluid, surface topography, sliding friction

## Abstract

Virtual surface characteristics of tactile displays are investigated to characterize the feeling of human touch for a haptic interface application. In order to represent the tactile feeling, a prototype tactile display incorporating Magneto-Rheological (MR) fluid has been developed. Tactile display devices simulate the finger’s skin to feel the sensations of contact such as compliance, friction, and topography of the surface. Thus, the tactile display can provide information on the surface of an organic tissue to the surgeon in virtual reality. In order to investigate the compliance feeling of a human finger’s touch, normal force responses of a tactile display under various magnetic fields have been assessed. Also, shearing friction force responses of the tactile display are investigated to simulate the action of finger dragging on the surface. Moreover, different matrix arrays of magnetic poles are applied to form the virtual surface topography. From the results, different tactile feelings are observed according to the applied magnetic field strength as well as the arrays of magnetic poles combinations. This research presents a smart tactile display technology for virtual surfaces.

## 1. Introduction

A haptic interface is a device used to probe virtual objects or environments without human interaction. The main haptic interface research areas are biological psychology, haptic interface design and control, haptic rendering and application. As virtual reality is requiring more realistic tactile perceptions for successful operations by haptic interfaces, more advanced techniques in the haptic interface design and control are needed to represent actual tactile feelings [1]. A large number of various actuating mechanisms can present sensations of shapes and textures. Among them, pressure based tactile displays with pin-arrays utilizing piezoelectric actuators is one of the more popular mechanisms [2]. Shape memory alloys [3], piezoelectric ceramics [4], ionic conductive polymer gel films [5], polymer fabrics [6] and electric motors [7] have been adopted as actuating devices in tactile display techniques. The electro-rheological fluid technique, which provides an inexpensive alternative to the other technologies because of the simple and flexible designs, has also been developed [8].

Similarly, as rheological properties of magneto-rheological (MR) fluid can change with magnetic field, a haptic display for surgical training in minimally invasive surgery applications has been realized based on a MR fluid [9]. The magneto-rheological fluid technique can represent the topography, compliance, and sliding feeling of flexible objects, such as tissues and tumors in biological applications. Since then, many investigations have continued to focus on the MR fluid applied in these applications. Scilingo *et al*. devised a haptic display able to mimic the compliance of biological tissues manipulated by surgical tools [10]. Then the authors established a simplified form of haptic black box based on the MR fluids technique [11]. Furthermore, they proposed two prototype of haptic displays for pinch-grasp and whole-hand exploration [12]. In another line of research, Liu *et al.* investigated the surface force response of a MR fluid-based haptic display with different electro-magnets in single cells, and their work provided a preliminary basis for future developments of this area [13].

A multi-cell haptic display using a magneto-rheological (MR) fluid is presented in this paper. In order to represent the sensations of contact such as the shape, compliance, and sliding (frictional) resistance, different tribological perceptions of the MR fluid are investigated by monitoring the normal force as well as the shearing force responses under controlled magnetic fields and arrayed magnetic poles. Thus, this work provides a continuation of research to develop tactile displays in multi-cells and its tribological aspects with the minimum number of mechanical components.

## 2. MR Fluid

MR fluids are typically suspensions of conductive particles in suitable nonconductive carrier liquids. When the fluid is subjected to an applied magnetic field, it will transform from a free-flowing liquid-like state into a solid-like state. At the same time, the rheological properties of the MR fluids will undergo instantaneous changes, such as an improvement of yield stress, shear viscosity and storage modulus with external magnetic field strength [14,15]. Interest in MR fluids derives from their ability to provide simple, quite rapid-response interfaces between electronic controls and mechanical systems [16]. MR fluids can be applied to various automotive, aerospace and industrial applications. For example, there are several types of the commercial MR shock absorbers and clutches [16,17]. A MR fluid changes from a Newtonian fluid in which particles move freely to a fluid displaying Bingham behavior in which particles are aligned in a chain, thus a variable yielding strength appears and can be controlled by the applied magnetic field. MR fluid particles are primarily micro-scale and are too dense to keep them suspended.

**Figure 1.**
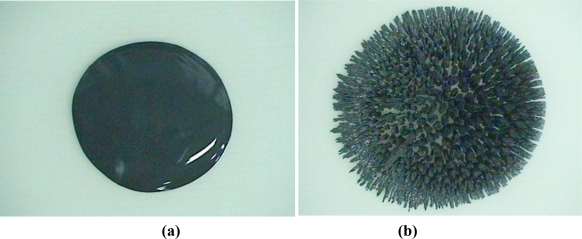
MR Fluid: **(a)** Liquid Phase. **(b)** Solid Phase.

**Figure 2.**
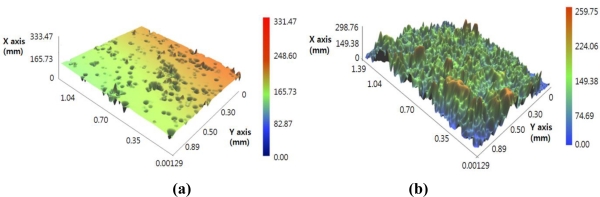
Microscopic surface response: **(a)** 45 G case. **(b)** 120 G case.

Figure 1 presents photographs of the surface changes of a MR fluid subjected to a magnetic field. Figure 1(a,b) shows the liquid phase under no magnetic field and the solid phase under a magnetic field, respectively. By using a noncontact surface profiler (NV-1000, Nano System), the surface roughness of MR fluid subjected to the specified magnetic field was measured. Figure 2(a,b) shows the microphotographs of the MR fluid subjected to magnetic fields. The surface roughness measured for the 45 G and 120 G cases are R_a_ = 4.83 μm, R_q_ = 12.37 μm, and R_a_ = 56.06 μm, R_q_ = 66.40 μm, respectively. Thus, the result shows the effectiveness of an MR fluid in application to tactile displays. In this work, the MRF-122EG MR fluid from LORD Corporation was used for the display, because of its economy and low viscosity which can show force response of magnetic field obviously. The material properties of the MR fluid are presented in Table 1.

## 3. Experimental

### 3.1. Experimental Apparatus

**Figure 3.**
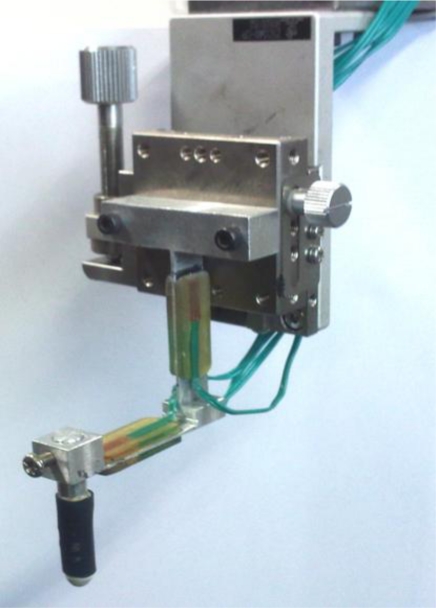
Monitoring device with dual strain gages and sensor tip.

**Figure 4.**
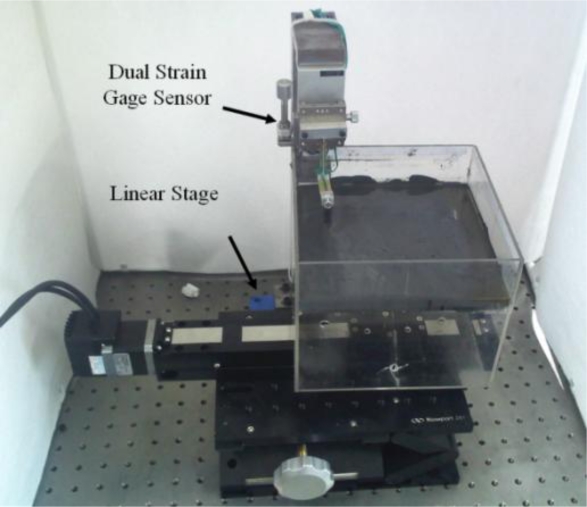
Photograph of experimental apparatus for MR haptic display.

In remote surgery, the surgeon should be able to use surgical instruments to interact with biological tissues and organs during an operation. Therefore, the tactile display has to be designed such that the operator can freely move the devices. In our experiments, the MR tactile box was developed using an acrylic material and the box size was 160 mm width, 160 mm length and 50 mm height. A module that applied the magnetic field was placed in the space under the tactile box. The proposed experiments focused on the measurement of the normal (vertical) and shearing (frictional) force responses of the MR fluid. Therefore, a monitoring device, designed using the dual stain gages, was constructed to sense the normal and shearing force responses, respectively. Output signals were sent to the computer for data analysis. Figure 3 presents the monitoring device with dual strain gages and a sensor tip, which directly contacts MR fluid. The designed sensor tip is made of aluminum 1,050. The size of the tip is 6 mm diameter and 40 mm length. In order to simulate the operation condition of an actual surgery, rounded rubber, which has radius of 3 mm, was attached in front of tip, and tip was covered with a latex glove. Figure 4 shows the experimental apparatus on the linear stage, which is operated by step motor. This linear stage is fully controlled by a computer to carry out operating motions.

### 3.2. Experimental Procedure

**Figure 5.**
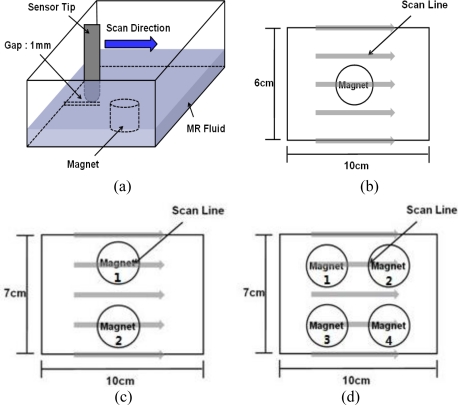
Procedure of experiments: **(a)** scan direction. **(b)** experiment I. **(c)** experiment II. **(d)** experiment III.

In order to minimize the size of the haptic display and apply a strong magnetic field under limited space, a permanent neodymium magnet was used in this research. The magnet is cylindrical, of 25 mm diameter and 30 mm height. The surface magnetic field flux, measured with a gauss-meter (Model-410, Brockhaus), was 0.552 Tesla (T). Figure 5 shows the procedure for experiments I, II and III.

Figure 5(a) depicts a 1 mm gap between the sensor tip and the bottom of the box, and the scan direction for the measurement. Experiment I, which is shown in Figure 5(b), was the single cell test for observing the tactile response of the MR fluid under different magnetic fields. A permanent magnet was placed at the center of the MR fluid box. In order to differentiate the magnetic flux density, a single magnet and double magnets were used whereby the surface magnetic flux density increased up to 0.604 Tesla. In order to monitor the virtual topography of the haptic display for single cell, 13 linear scans were conducted for 6 cm scan width, 10 cm scan length, at 0.1 mm/sec scan speed. Experiment II, shown in Figure 5(c), is the test with an array of magnetic poles for observing tactile feeling changes under different array combinations in a row. For experiment II, 17 linear scans were conducted for line width of 7 cm, scan length of 10 cm, and scan speed of 0.1 mm/s. Experiment III depicted in Figure 5(d) is the test for a 2 × 2 matrix array of magnetic poles and cases. The experiments for experiment III were conducted with 17 linear scan lines, line width 7 cm, scan length 10 cm, and scan speed 0.1 mm/s. All the combination conditions of magnets are shown in Table 2. The environment condition of pressure was 1 atm. The temperature was 21.2 °C and the relative humidity was 46%.

## 4. Results and Discussion

**Figure 6.**
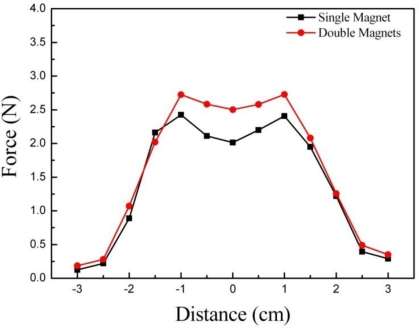
Normal force response of experiment I.

**Figure 7.**
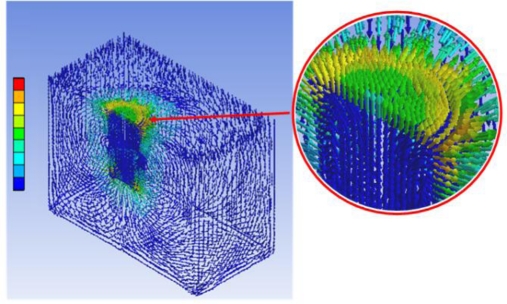
Magneto-static simulation result.

**Figure 8.**
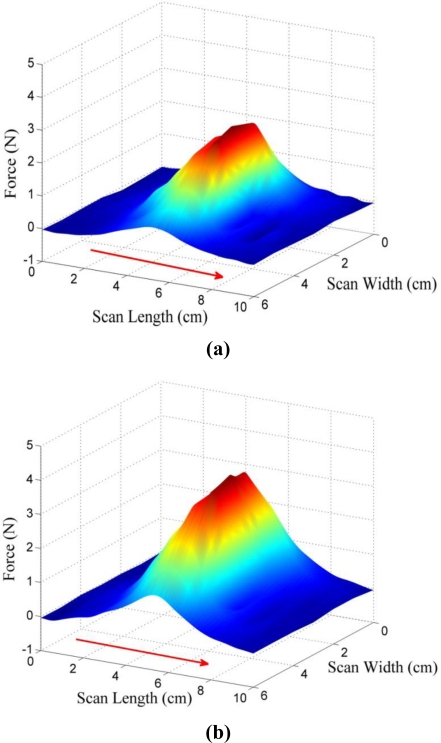
Shearing force responses in experiment I: **(a)** single magnet (0.552T). **(b)** double magnets (0.604T).

Figure 6 shows the normal (vertical) force response for experiment I. Experiments were conducted using a single magnet and double magnets. As shown by the results, double magnets can generate a higher response force (maximum 2.8 N) than a single magnet (maximum 2.4 N). The results clearly show that different vertical force responses for different magnetic fields can be expressed by using the MR haptic display. The magneto-static simulation of the haptic display was conducted using ANSYS^TM^ with a single cell to explain the dimples both shown at the centers of the graphs in Figure 6. Figure 7 presents the result of the simulation. It clearly shows the low magnetic flux density at the center of display, which is similar to the result of the experiments. Shearing (frictional) force responses for experiment I are shown in Figure 8. As shown in the results, the maximum shearing force response is increased from 2.83 N (single magnet) to 3.86 N (double magnets) with the increase of the magnetic field density. Also the size of the magnets used can affect the shape and height of the peak point of the shearing force response. Thus, the results apparently explain how finger sensation can be changed during an operation by controlling the magnetic field.

**Figure 9.**
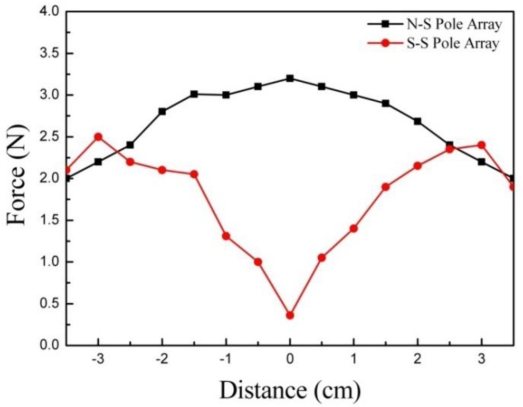
Normal force response of experiment II (Single magnet).

Figure 9 shows the normal force responses for experiment II, where the test procedure is explained in Figure 5(c). As shown in the graphs, two totally different behaviors are observed under different magnetic pole arrays. Test case II-1 (N-S pole array) maximized the magnitude of the normal force response to 3.2 N, whereas test case II-2 (N-N pole array) minimized the magnitude of the normal force response at center to 0.4 N. Thus, the N-S pole array placed at two locations on the magnet is more effective in increasing the normal force response than the double magnets placed at the center. Also, the N-S pole array eliminates the dimple at the center of the display, which is shown in Figure 6.

**Figure 10.**
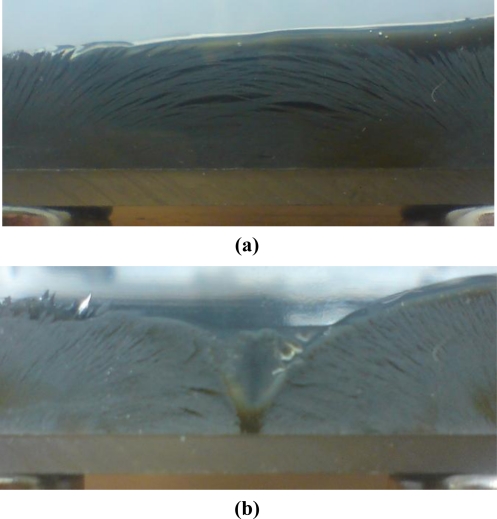
Photographs of experiment II: **(a)** N-S (opposite) pole array. **(b)** S-S (same) pole array.

**Figure 11.**
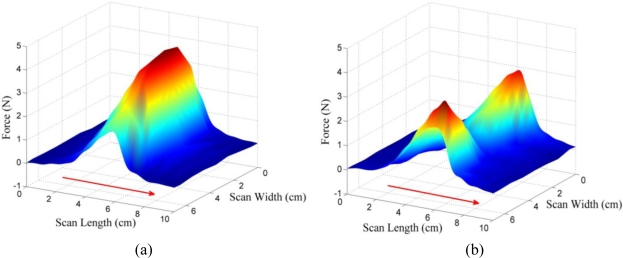
Shearing force responses of experiment II: **(a)** N-S (opposite) pole array. **(b)** S-S (same) pole array.

Figure 10 presents photographs of experiment II, which show the actual behavior of the MR fluid for different pole arrays. As shown in Figure 10(a), particles in the MR fluid are aligned like a bridge between the poles under the opposite (N-S) pole array. However, as shown in Figure 10(b), the particles in the MR fluid are disconnected between two poles under the same (S-S) pole array. The photographs clearly show that the MR particles are aligned in the direction of the magnetic flux. Figure 11 depicts the shearing force responses of experiment II in 3-dimensional view as well as in scan-width view. Showing the same trend as the normal force response, the result of test case II-1 (N-S pole array) shows the generation of the maximum shearing force response of 4.5 N, whereas that of test case II-2 (S-S pole array) shows the generation of the minimum shearing force of 0.5 N at the center of the display. The opposite pole array of magnets [Figure 11(a)] also generates an even greater shearing force response than the double magnets do at the center [Figure 8 (b)]. From the experimental results, an MR haptic display with the different combinations of magnetic poles can express the virtual surface curvature of an internal organ.

**Figure 12.**
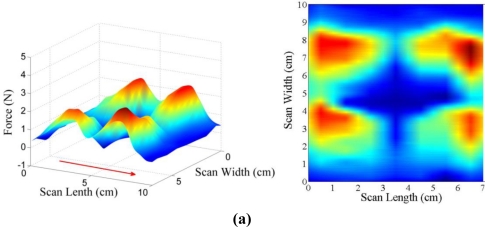

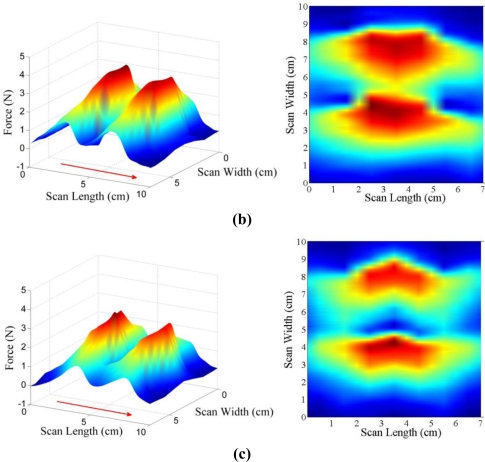
Shearing force responses of experiment III: **(a)** case III-1 [S Pole, S Pole; S Pole, S Pole]. **(b)** case III-2 [S Pole, S Pole; N Pole, N Pole]. **(c)** case III-3 [S Pole, N Pole; N Pole, S Pole].

In order to investigate the effect of the matrix array of magnetic poles on the tactile frictional feeling, the shearing force responses of experiment III are shown in a 3-dimensional view as well as at the view from the top. Three test cases were examined, and detail experimental procedures are explained in Table 2 and Figure 5(d). As shown in Figure 12(a), all the same magnetic poles in the matrix array generate the deepest dimple at the center of the display, and this phenomenon is also found in Figure 11(b) (S-S same pole array). However, the peak values in Figure 12(a) are not as high as the other peak values in Figure 12(b,c), which both show the results for the opposite pole arrays, but case III-2 in Figure 12(b) produces a little higher maximum shearing force response than case III-3 in Figure 12(c). Furthermore the shape of the peaks is totally different: the peaks for case III-3 [Figure 12(d)] are sharper than those for case III-2 [Figure 12(c)] due to the cross pole positions in the matrix array of case III-3. From the experiment, it is found that a more complex expression of the virtual surface can be obtained using the magnet combinations in the matrix array. Thus, the results also proved the feasibility of representing virtual 3-dimensional topography using the MR haptic display.

## 5. Conclusions

A tactile display module using an MR fluid has been developed in this research to evaluate virtual surface characteristics. The display takes advantage of the fact that MR fluids display different rheological (stiffness) properties as well as different surface roughness under various magnetic fields. In order to demonstrate the sensations of the finger on the virtual surface, normal force responses as well as shearing force responses are monitored using the designed monitoring device with dual strain gages and a sensor tip. An MR haptic display module was prepared to measure the force responses under different magnetic flux densities and array positions. From experiment I, it was found that the maximum normal force as well as the shearing force is increased by increasing the magnetic flux density. Also, the size of magnet itself can affect the shape and height of the peak of the force responses. Experiment II showed that the proposed MR haptic display with different combinations of magnetic poles can express the virtual surface curvature of objects. Finally, the results of experiment III confirmed the feasibility of expressing 3-dimensional virtual surfaces using magnet combinations in matrix array. Tactile sensations of the virtual surface may be expressed by arranging the electromagnets in complex combinations.

## Figures and Tables

**Table 1.** Properties of the MR Fluid.

**Properties**	**Value**
Viscosity, Pa-s @ 40 °C	0.042 ± 0.020
Density(g/cm^3^)	2.28 to 2.48
Operating Temperature, °C	−40 to + 130

**Table 2.** Experiment Conditions.

**Experiment Number**	**Case Number**	**Magnet pole arrays**
Experiment I^1)^	Case I	Single Magnet
	Case II	Double Magnets
Experiment II^2)^	Case I	1 (N Pole); 2 (S Pole)
	Case II	1 (S Pole); 2 (S Pole)
Experiment III^3)^	Case I	1 (S Pole); 2 (S Pole); 3 (S Pole); 4 (S Pole)
	Case II	1 (S Pole); 2 (S Pole); 3 (N Pole); 4 (N Pole)
	Case III	1 (S Pole); 2 (N Pole); 3 (N Pole); 4 (S Pole)

(1), (2), (3) are related to Figure 5(b), Figure 5(c) and Figure 5(d), respectively.
